# Metagenomic insights into traditional fermentation of rice-based beverages among ethnic tribes in southern Assam, Northeast India

**DOI:** 10.3389/fmicb.2024.1410098

**Published:** 2024-09-11

**Authors:** Hanna Yumnam, Parijat Hazarika, Indu Sharma

**Affiliations:** ^1^Department of Microbiology, Faculty of Science, Assam University, Silchar, India; ^2^Programme of Microbiology, Faculty of Science, Assam down town University, Guwahati, India

**Keywords:** fermented foods, microbiome communities, high-throughput sequencing, Iluminia MiSeq, rice-based beverages, ethnic tribes

## Abstract

**Introduction:**

Traditional fermented foods have long been recognized for their numerous health benefits along with their potential to aid in the treatment of gastrointestinal disorders. These fermented foods have been shown to promote gut health and contribute to a longer, healthier life.

**Methods:**

The high-throughput sequencing using the Illumina MiSeq platform was employed to investigate the microbiome communities of rice-based fermented beverages consumed by ethnic tribes in Southern Assam, namely Zeme Naga, Dimasa Kachari, Hmar, Karbi and Tea tribes.

**Results:**

The fermented rice-based beverages were highly predominated by *Firmicutes, Bacteroides, Proteobacteria*, and *Actinobacteria* exhibiting the highest relative abundance across all tribes. At genus level, significant abundance of *pediococcus, lactobacillus, bacillus, leuconostoc, acetobacter, staphylococcus, delftia, erwinia, klebsiella* and *chrysebacterium* were found amongst these ethnic tribes.

**Discussion:**

Understanding the fermented food microbiome will help to know the relationships between microbial communities and their effect on health of humans amongst the tribes. Furthermore, the use of these fermented products could provide enhanced health benefits to southern Assam region of India.

## 1 Introduction

Ancient cultures all over the world have been preparing traditional fermented foods, which have a number of health advantages. Through their metabolic processes, a variety of advantageous microbes endow final fermented food products with special qualities that enhance nutrition, lower endogenous toxins, and increase digestibility by breaking down proteins in meals to produce organic acids, providing probiotic benefits and preserving the foods for a prolonged period (Sekar and Kandavel, 2002). Therefore, elucidating the diversity and abundance of bacteria in fermented foods is of great importance to understanding how microbial taxa contribute to flavor, taste, and other health benefits (Tsafrakidou et al., 2020; Deka et al., 2021). Fermented foods are gaining popularity because of their distinctive flavors, nutritional value, and favorable organoleptic features (Marco et al., 2017). The population of Northeastern India has a rich heritage of preparing traditional fermented products, deeply rooted in ethnic wisdom that has been passed down through generations for numerous years (Das A. et al., 2012; Das A. J. et al., 2012; Rawat et al., 2018). The ethnic tribes of southern Assam in Northeastern (NE) India have used their native rice-based fermented foods since time immemorial. Every ethnic tribal population has its own ethnic fermented food and beverages. However, different tribes prepare their own traditional rice-based beverages based on the accessibility of food substrates available in the environment to which they belong. These food items improve microbial stability, digestibility, detoxification, and nutrition content, all of which are beneficial to human health (Tamang et al., 2012). However, as fermented foods are important for maintaining the balance and regulation of the intestinal microbiota, their significance as probiotics is steadily growing (Marco et al., 2017). An ethnic group’s identity can be discerned largely through its cuisine (Keishing and Banu, 2013). Most ethnic fermented foods and alcoholic beverages used by the various ethnic tribal people in the northeastern states of India are exclusive to a particular location and are prepared using unique techniques that involve the use of diverse substrates (Tamang et al., 2012; Keishing and Banu, 2013; Das et al., 2016). Similarly, in southern Assam, diverse ethnic communities also have a rich tradition of consuming rice-based fermented beverages, which have become an important part of the diet and cultural heritage. For instance, the Zeme Naga tribes brew a locally rice-based fermented beverage called ‘Zao,’ with a starter culture called ‘Ndhuihi,’ and the Dimasa Kachari make ‘Judima,’ a rice-based beverage, using a starter culture called ‘humao.’ The Hmar tribes prepare their local rice-based beverage ‘Zu’ with a starter culture called ‘Chol’, while the Karbi tribes prepare ‘Hor Alank’ with a starter culture known as ‘thap,’ and the Tea tribes, prepare ‘Haria,’ a common rice-based fermented beverage with a starter culture called ‘dawai’. Glutinous local rice is favored for its preparation, where the rice is washed, cooked, and mixed with the starter culture. Then, the mixture undergoes natural fermentation for 4–5 days to a week in earthen pots covered with banana leaves or sacks. The liquid is then filtered after fermentation and transferred to another vessel, ready for consumption. The process of making rice-based beverages is somehow similar; each group uses distinct local ingredients for their preparation of starter culture. This traditional practice not only reflects cultural diversity but also garners academic attention for its unique preparation methods and community significance. Each tribe has developed its own techniques of preparation based on the availability of resources, contributing to the diversity of these fermented products.

Fermented foods and beverages are abundant in the northeastern states of India, yet there is a significant lack of documentation about these products. The impact of globalization on these traditional items in the region remains largely undocumented. Additionally, there is an absence of comprehensive research on the traditional food processing and preservation techniques specific to the southern Assam area. Importantly, the application of advanced scientific methodologies and techniques in the production of fermented foods and beverages has not been thoroughly studied. Next-generation sequencing (NGS) techniques have recently made advancements that have given us platforms to investigate microbial activity and diversity in a range of situations (Mehetre et al., 2016; Cao et al., 2017). Considering the enormous range of fermented products in the northeastern states of India, very few studies related to NGS technology have been performed on fermented foods that are native to this region (Deka et al., 2021). Henceforth, the scope of our study is to characterize the microbial populations present in rice-based fermented products from five distinct ethnic tribes in southern Assam: the Zeme Naga, Dimasa Kachari, Hmar, Karbi, and Tea tribes using advanced next-generation sequencing (NGS) techniques, particularly the Illumina MiSeq platform for high-resolution analysis and bioinformatics analysis based on the V3–V4 region of the 16S rRNA gene.

## 2 Materials and methods

### 2.1 Sample size

A total of 15 samples were collected using traditional sampling protocols, excluding commercially sold rice-based fermented products available in the local markets. Only those products prepared by the ethnic communities using traditional methods were included, namely ‘Zao’ by Zeme Naga, ‘Judima’ by Dimasa Kachari, ‘Zu’ by Hmar, ‘Hor Alank’ by Karbi, and ‘Haria’ by Tea tribes. The samples were taken prior to distillation, collected in sterilized vials, transported to the laboratory, and stored in the refrigerator at 4°C.

### 2.2 Study design

The traditional methods for preparing rice-based fermented products were documented, along with the collection of samples from five distinct ethnic groups belonging to the Zema Naga, Dimasa Kachari, Hmar, Karbi, and Tea tribes of southern Assam, India, based on information provided by respondents from each group. Out of a total of 15 samples, three samples were collected from each ethnic community cohort.

### 2.3 Quantitative biochemical analysis of the sample

To assess the quantitative biochemical analysis, pH and electric conductivity (EC) of the collected samples were determined using a digital pH meter (Sartorius AG Germany) and a Deluxe Water and Soil Analysis Kit (Model LT-60) manufactured by Labtronics, India, respectively.

### 2.4 Microbial harvesting and DNA isolation

The beverage samples were centrifuged at 3,200 × *g* for 20 min at 4°C, and the resulting supernatant was removed. The pellet was then reconstituted in lysis buffer, heated at 65°C for 10 min, and subsequently processed for DNA isolation. The QIAamp PowerFecal Pro DNA Kit (Catalog No. 51804) (QIAGEN, Hilden, Germany) was used to extract DNA according to the manufacturer’s instructions. The extracted DNA samples were quantified on NanoDrop One and Qubit using water and standards as a control, respectively. Based on QC results, 16S V3–V4 PCR was performed on all the samples using either 12.5 ng of DNA quantified by Qubit or 10 μL of DNA (for low concentration). Finally, the DNA amplicons were stored at −20°C for further sequencing (Lim et al., 2020).

### 2.5 Metagenomic sequencing

The collected samples were analyzed using the Illumina MiSeq Platform to sequence the V3 and V4 regions of the bacterial 16S rRNA gene. The 16S metagenomic library of the DNA from rice-based fermented beverages was prepared using region-specific primers targeting the V3–V4 region of the bacterial 16S rRNA (Yang et al., 2018). Each sample was uniquely identified through indexing PCR using an Illumina NextEra XT Index kit. The library, with an estimated amplicon size ranging from approximately 480 to 530 base pairs, was purified using Agencourt AMPure XP SPRI beads (Beckman Coulter, California, USA) to ensure the removal of undesired fragments and to optimize DNA quality, and the library was quantified using a NanoDrop spectrophotometer (Life Technologies, USA). The index PCR products of each library were combined in equimolar concentrations into a single pool. The quantification of the pooled library was conducted using a high-sensitivity DNA chip present in the bioanalyzer (Agilent Technologies) after gel purification. Sequencing was carried out using a 500-cycle reagent kit on the Illumina MiSeq platform as per the manufacturer guidelines, enabling the generation of clusters and paired-end sequencing reads for comprehensive analysis of the bacterial communities within the fermented beverages (Dubey et al., 2018; Hazarika et al., 2022).

### 2.6 Bioinformatics and statistical analysis

Illumina MiSeq platform-produced paired-end V3–V4 reads were demultiplexed using the Bcl2Fastq tool. Sequence readings were further assessed based on their quality score using FASTQC^1^ (Cock et al., 2010). For further analysis, only relevant, high-quality reads that underwent a Fastq join were chosen (Caporaso et al., 2010). The study excluded sequences with ambiguous bases, homopolymer runs of more than 6, primer mismatches, or lengths of less than 100 base pairs. Using the software program QIIME (Quantitative Insights into Microbial Ecology), additional processing was applied to the remaining readings (Caporaso et al., 2010). Operational taxonomic units (OTUs) were determined at a 97% sequence identity threshold using the May 2013 Greennenes database (v 13.8), and based on the OTUs’ 97% similarity to reference sequences, taxonomy was assigned to them (DeSantis et al., 2006). The 16S rRNA database was painstakingly vetted and free of chimeras, and query sequences were clustered using the UCLUST (Usearch clustering) method (Edgar, 2010). Taxonomic distribution summaries, ranging from phylum to genus, were then generated using the OTU table derived from this analysis, without rarefaction. A biome file was generated to facilitate further analysis. It is a structured dataset used in microbiome research to consolidate information on microbial community composition and predicted functions. Clustering was conducted based on normalized OTU abundances, eliminating OTUs that accounted for less than 0.005% of the total sequence. To assess diversity among individuals, alpha-diversity metrics (the Shannon index and Simpson index), beta diversity, and principal coordinate analysis (PCoA) were calculated using QIIME. Functional analysis was performed using the PICRUSt tool (Phylogenetic Investigation of Communities by Reconstruction of Unobserved States), which generated a biome file. The functional compositions of diverse microbial communities were analyzed using the Kyoto Encyclopedia of Genes and Genomes (KEGG) database (Kanehisa and Goto, 2000), which is accessible to academic users at https://www.kegg.jp/. Programs such as ‘Adonis’ were used to perform the permutational multivariate analysis of variance, (PERMANOVA) (weighted and unweighted UniFrac distance matrix). The ratio of the observed OTU frequencies in the sample groups to the expected frequencies based on the extrinsic hypothesis was compared using the parametric G test to evaluate if there was a statistically significant difference in the abundance of OTUs in the various sample groups. Taxa abundance comparisons between different cohorts were performed using the Kruskal–Wallis H-test (Kanehisa et al., 2016; Hazarika et al., 2022). All the statistical analyses were performed using R version 3.6.2. We used the ‘vegan’ R package^2^ (Huson et al., 2007) to measure the microbial richness, evenness, and diversity.

## 3 Results

### 3.1 Demographic characteristics

The traditional beverage preparation practices of each ethnic tribe involve the use of rice as a substrate, which is mixed with native plants that act as a starter culture (Supplementary Figure S1). The fermentation process lasts up to a week, depending on seasonal conditions. A comprehensive summary of the specific preparation conditions employed by each ethnic tribe is presented in Table 1, highlighting the specific aspects of their traditional beverage production methods. The demographic features of the ethnic tribes are represented in Table 2, highlighting their population, geographic distribution, and cultural practices.

**Table 1:** The specific conditions under which the beverages were prepared by each ethnic group.

Ethnic groups	Beverages	Conditions prepared
Zeme Naga	Zou	Rice and indigenous plants are used, Fermentation takes 3–5 days (Atungbou, 2020)
Dimasa Kachari	Judima	Rice and indigenous plants are used, 3–5 days for fermentation (Arjun et al., 2014)
Hmar	Zu	Rice and indigenous plants are used, 3–5 days depending on the seasons
Karbi	Hor Alank	Rice and indigenous plants are used, 2–3 days depending on seasonal conditions (Borah et al., 2021)
Tea tribes	Haria	Rice and indigenous plants are used, fermentation takes up to 3–4 days (Ghosh et al., 2014)

**Table 2:** Demographic features of ethnic groups.

Tribes	Population ^a^	Distribution ^a^	Cultural practice
Zeme Naga	Approximately10,000	Cachar	Fermentation, weaving, handicrafts, livestock, and consumption of meats (Longkumer, 2016)
Dimasa Kachari	Approximately 52,482	Cachar, Hailakandi, and Karimganj	Weaving, jhum cultivation, handicrafts, and fermentation (Konwar et al., 2023)
Hmar	Approximately 19,797	Cachar and Karimganj	Fermentation of food and beverage, livestock, cultivation of paddy, crops and vegetables, and meat consumption
Karbi	Approximately 22,194	Karimganj	Animism, jhum cultivation, agriculture, and livestock (Kropi, 2016)
Tea tribes	Approximately 1,00,102	Cachar, Hailakandi, and Karimganj	Tea gardening and food habits include traditional Assamese sweets (Magar and Kar, 2016)

^a^According to the Director of Census Operations, Assam, India (https://www.censusindia2011.com/), the mentioned distribution is specific only to the places where the study included these ethnic groups within southern Assam.

### 3.2 Biochemical analysis

The biochemical analysis of traditionally rice-based fermented beverages was evaluated for pH and electric conductivity (EC) and expressed as mean ± standard deviation (SD) value (Table 3). The fermented rice-based beverages collected from all five distinct ethnic tribes exhibited a pH range of 3 to 4 and electrical conductivity between 0.5 and 1. These values are significant indicators of the biochemical processes occurring during fermentation. The pH range reflects the acidity level of the fermented beverage, which is crucial for the growth of bacterial strains responsible for fermentation, while the electrical conductivity values observed may indicate the concentration of dissolved ions, such as salts and organic acids, which may contribute to the drink’s flavor profile and microbial stability. The similar values observed across tribes suggest shares brewing practices possibly influenced by local microbial ecology and cultural traditions.

**Table 3:** pH values and electric conductivity (EC) measurements of rice-based beverage samples.

Sample ID	pH	EC (S/cm)
DMK	3.6 ± 0.25	0.65 ± 0.06
HM	3.4 ± 0.21	0.86 ± 0.05
K1	3.5 ± 0.27	0.75 ± 0.1
ZN	3.5 ± 0.24	0.8 ± 0.09
TT	3.5 ± 0.3	0.6 ± 0.08

### 3.3 Statistics of sequencing data

**Figure 1:**
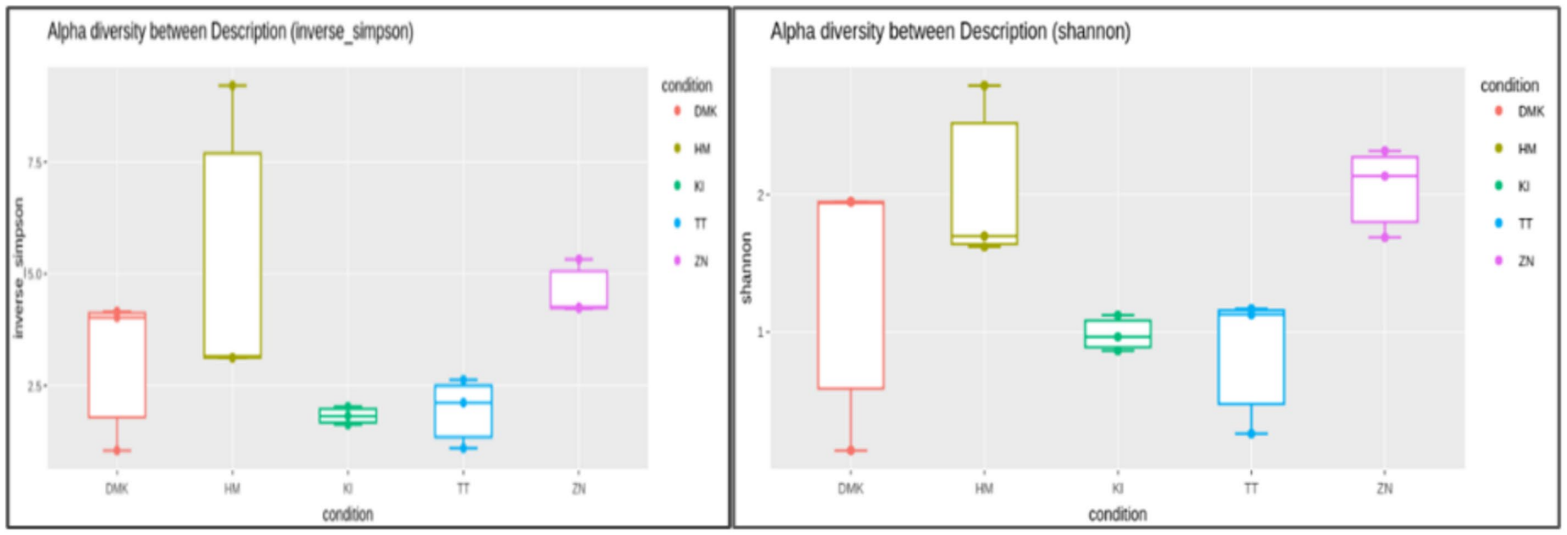
Alpha diversity analysis using the inverse Simpson index and Shannon index for rice-based beverages among all the five ethnic groups. Values show diversity metrics: Zeme Naga tribe (4.2; 2.2); Dimasa Kachari tribe (3.9; 1.8); Hmar tribe (2.9; 1.6), Karbi tribe (1.4; 0.9), and Tea tribe (1.8; 1.3). Higher values indicate greater microbial diversity within the fermented beverage of each group.

**Figure 2:**
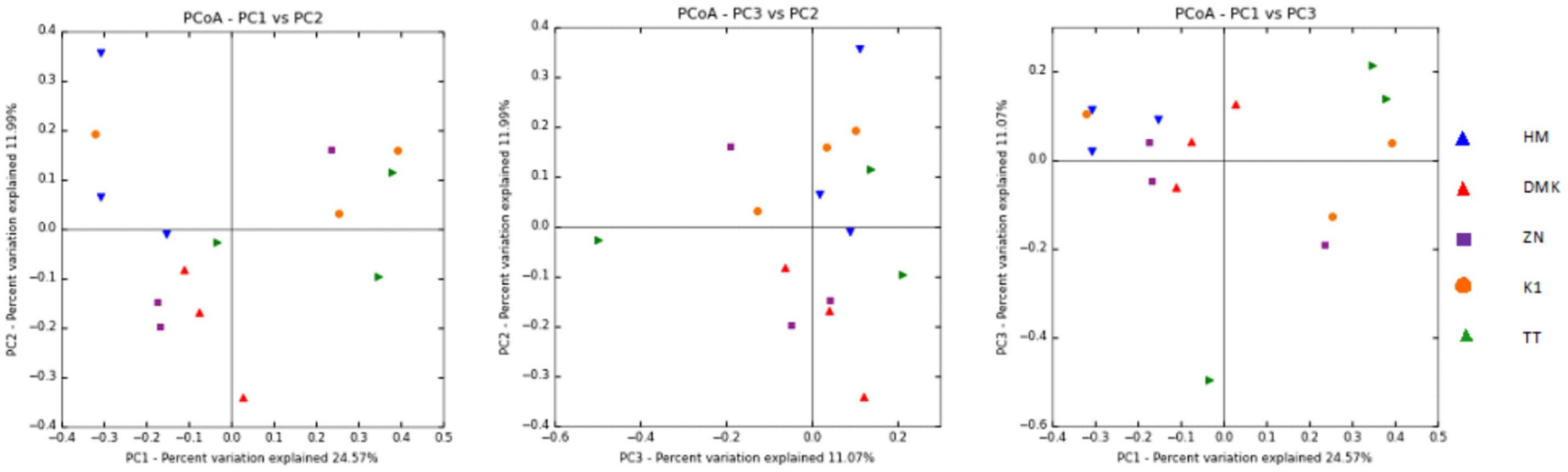
Principal Coordinate Analysis (PCoA) was plotted, which showed the similarities between the microbial communities present in rice-based fermented beverages derived from the five ethnic tribes (Zeme Naga, Dimasa Kachari, Hmar, Karbi, and Tea tribes).

In this present study of rice-based fermented beverages among five ethnic groups, we utilized the Cumulative Sum Scaling (CSS) normalization method to account for differences in sequencing depth and compositional biases. The read count statistics varied significantly across the tribes: the Zeme Naga tribe ranged from 16,603 to 137,757 reads, the Dimasa Kachari tribe ranged from 22,652 to 45,696 reads, the Hmar tribe ranged from 43,194 to 113,683 reads, the Karbi tribe ranged from 119,306 to 144,316 reads, and the Tea tribe ranged from 74,487 to 190,837 reads. These wide ranges reflect the diverse sequencing depths observed in our samples. Alpha diversity analysis of the five studied communities for rice-based beverages showed significant differences among the tribes using the inverse Simpson diversity index (*p* < 0.05) and Shannon index (*p* < 0.05) (Figure 1). The Kruskal–Wallis rank-sum test revealed higher diversity and abundance in the Zeme Naga (ZN) tribe, followed by the Dimasa Kachari (DMK) tribe, while lower abundance was observed among the Hmar (HM) tribe, the Karbi (K1) tribe, and the Tea tribe (TT). A beta diversity plot assessed by the unweighted UniFrac method revealed the similarity of microbiota compositions among the five tribes, which was statistically significant (*p* > 0.05). These findings underscore substantial variations in microbial composition across the different tribes’ rice-based fermented products, influenced by cultural practices, environmental factors, and possibly genetic diversity. The principal coordinate analysis (PCoA) of rice-fermented foods revealed variable microbiota, with Principal Component 1 (PC1) explaining 24.57% of the variation, followed by PC2 with 11.99% and PC3 with 11.07%. PCoA provides insights into the similarity among samples from different tribes based on the composition of observed microorganisms (Figure 2).

### 3.4 Bacterial taxonomic composition across five ethnic groups

**Figure 3:**
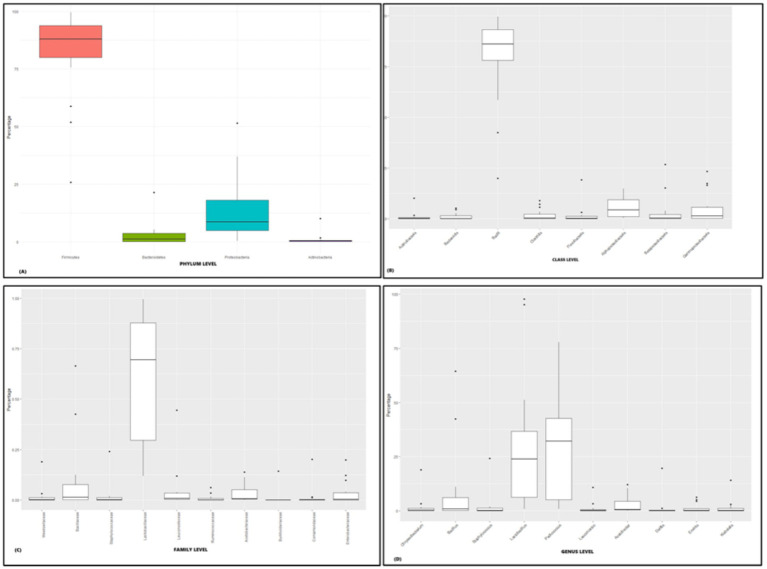
Relative abundance of major taxonomic groups in rice-based fermented beverages brewed by five ethnic tribes in southern Assam: **(A)** phylum-level analysis (*p*-value- 0.041), **(B)** class-level analysis (*p* – 0.024), **(C)** family-level analysis (*p* – 0.014), and **(D)** genus-level analysis (*p* – 0.001).

The microbial composition of rice-based fermented samples was examined across five ethnic tribes, analyzing the relative abundance at various taxonomic levels including phylum, family, class, and genus. Metagenomic analysis was employed to determine the composition of rice-based fermented microbiota in the Zeme Naga, Dimasa Kachari, Hmar, Karbi, and Tea tribes of southern Assam. Significant abundance of *Firmicutes, Proteobacteria, Actinobacteria,* and *Bacteroides* at the phylum level was observed among the five tribes (*p – 0.041*, Kruskal–Wallis *H*-test) (Figure 3A). At the class level, the predominant bacteria across the five ethnic tribes belonged to *Bacilli, Actinobacteria, Clostridia, Bacteroidia, Flavobacteriia, Betaproteobacteria, Alphaproteobacteria,* and *Gammaproteobacteria* (*p - 0.024*, Kruskal–Wallis *H*-test) (Figure 3B).

The most prevalent families identified among the five ethnic groups of southern Assam were *Lactobacillaceae, Bacillaceae, Leuconostoceae, Enterobacteriaceae, Acetobacteraceae, Ruminococcaceae, urkholderiaceae, Staphylococcaceae, Comamonadaceae,* and *Weeksellaceae* (*p* – 0.014, Kruskal–Wallis *H*-test) (Figure 3C). Significant differences in bacterial abundance were observed among the ethnic tribes at the genus level. The identified genera with notable abundance included *Lactobacillus, Bacillus, Pediococcus, Leuconostoc, Acetobacter, Delftia, Erwinia, Klebsiella, Chryseobacterium,* and *Staphylococcus* (*p – 0.001*, Kruskal–Wallis *H*-test) (Figure 3D). Permutational multivariate analysis of variance (PERMANOVA) analysis revealed that at the phylum level, the composition of microbial communities among these rice-based fermented beverages of five ethnic groups did not show a significant difference (PERMANOVA *F* statistic = 1.19, *p*-value = 0.2) (statistical significance, *p* > 0.05).

### 3.5 Functional profiles in rice-based fermented samples by PICRUSt analysis

**Figure 4:**
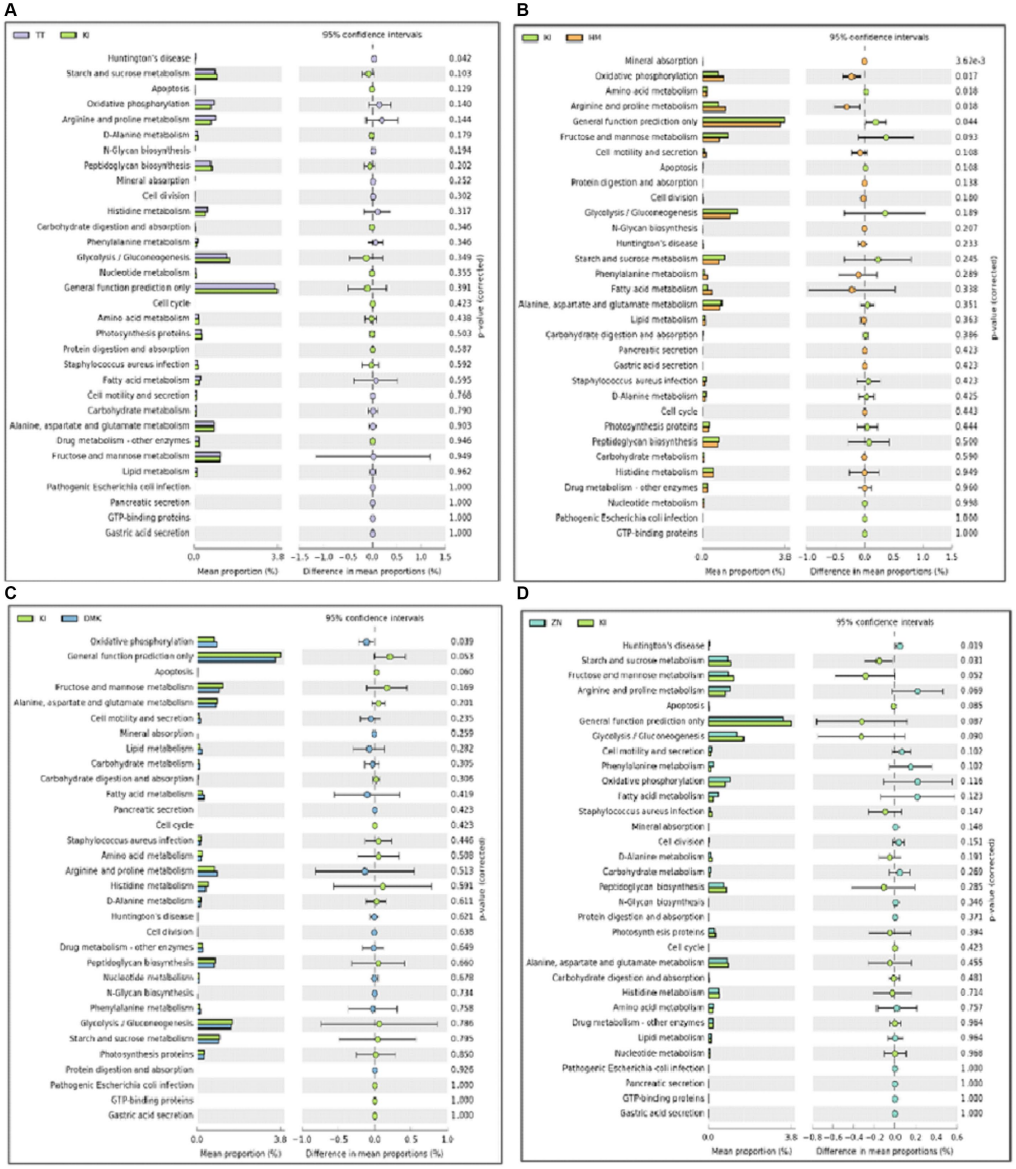
**(A–D)** A comparative analysis was conducted using PICRUSt (Phylogenetic Investigation of Communities by Reconstruction of Unobserved States) to evaluate the relative abundance of genes associated with functional features in the microbiota of rice-based fermented samples from ethnic tribes in southern Assam. The analysis highlights significant differences among the five study groups. These differences were identified at a 95% confidence level with a *p*-value < 0.05, indicating statistical significance.

The PICRUSt program (phylogenetic research of communities by reconstruction of unobserved states) was employed for a comprehensive grasp of the potential functional profiles of the microbiota found in samples of fermented rice-based products (Langille et al., 2013). Functional prediction analysis revealed that the rice-based fermented products of the Karbi (K1) tribe are closely related to various metabolic functions such as starch and sucrose metabolism, fructose and mannose metabolism, and general function prediction only. The products of the Hmar (HM) tribe show an abundance of genes related to oxidative phosphorylation and arginine and proline metabolism. The products of the Dimasa Kachari (DMK) tribe have genes related to oxidative phosphorylation (Figures 4A–D). Such variations in gene abundance among fermented samples can be attributed to several factors including genetic differences among the presence of microbial communities or unique microbial strains in each fermented product; environmental conditions such as temperature and pH, which may influence microbial growth and metabolism; cultural practices that dictate fermentation methods and ingredients used; and adaptation of the microbes to specific fermentation conditions and its duration over time, which may impact the extent of metabolic activities and gene expression profiles.

## 4 Discussion

Rice-based fermented beverages hold significant popularity among the diverse ethnic communities of Assam, where they are traditionally prepared and consumed during religious and harvest festivals (Bora et al., 2016). People have been practicing food fermentation for many years, which improves microbial composition and has a significant medicinal impact on health (Leech et al., 2020). The indigenous inhabitants of southern Assam, who live in remote villages with unspoiled surroundings, consume fermented rice-based drinks. The environmental conditions of southern Assam are very specific, such as variations in seasonal and climatic conditions and geographical locations, which play a significant role in shaping the composition of microbial communities present in rice-based fermentation. These factors may play a role in specific characteristics and unique potential variations in the rice-based beverage prepared by different ethnic populations in this region. Therefore, understanding these influences may aid in optimizing the production of local alcoholic beverages, emphasizing sustainable practices and local market demands, and also preserving the cultural heritage linked to these traditional beverages through their fermentation practices. However, knowledge of the microbial diversity of fermented rice-based beverages in such distinct and isolated parts of southern Assam in Northeastern India is limited. Henceforth, the present study analyzed the microbial composition of fermented rice-based beverage products belonging to the Zeme Naga, Dimasa Kachari, Hmar, Karbi, and Tea tribes of southern Assam.

In our present study, the bacterial composition of fermented rice-based beverages among the five ethnic tribes’ dominants by phylum was *Firmicutes,* followed by *Proteobacteria*, *Bacteroidetes*, and *Actinobacteria* with reduced relative content. A recent study showed (Zhao et al., 2022) that *Firmicutes* and *Proteobacteria* are significantly higher in rice wine from Koji, China. Similarly, a study on marcha and thiat, a starter culture used for the preparation of alcoholic beverages in Sikkim and Meghalaya states in India, revealed a predominance of *Proteobacteria*, followed by Firmicutes and Actinobacteria (Sha et al., 2017). In contrast to our findings, analysis of an ethnic rice wine starter culture (*Xai-pithai*) from Assam revealed a dominance of *Streptophyta,* followed by *Proteobacteria* and *Ascomycota* (Bora et al., 2016). These findings could be attributed to variations in environmental conditions across different seasons of production, influencing the abundance and composition of microbial diversity. Moreover, these microbial diversities are likely to have migrated from the starter culture and played pivotal roles in the fermentation process of beverages.

Various genera of *Enterococcus, Lactobacillus, Lactococcus, Leuconostoc, Pediococcus, Streptococcus, Vagococcus,* and *Weissella* have been isolated from fermented foods and beverages worldwide (Salminen et al., 2004; Axelsson et al., 2012; Holzapfel and Wood, 2014). The present study revealed the predominance of bacterial genera *Lactobacillus,* followed by *Pediococcus*, *Bacillus*, *Acetobacter,* and *Staphylococcus,* and a lower abundance of *Leuconostoc, Erwinia, Klebsiella, Delftia,* and *Chryseobacterium.* This microbial composition may contribute to anti-microbial and anti-inflammatory activity and also prevent nutritional losses by inhibiting the growth of spoilage bacteria. In North Bengal, India, genera such as *Bacillus, Arthrobacter, Lactobacillus, Clostridium,* and *Lactococcus* were predominantly found in rice-based fermented liquor among the Rabha tribe (Bhattacharjee et al., 2023). Similarly, a recent study (Zhao et al., 2022) stated that *Weissella* emerged as the dominant genus, followed by *Pediococcus*, *Lactobacillus*, *Leuconostoc*, *Acinetobacter*, *Klebsiella*, *Staphylococcus*, *Bacillus*, *Enterobacter,* and *Cronobacter,* which is quite similar to our findings. Interestingly, a study conducted across various regions of China using rice koji observed a high prevalence of *Lactobacillus, Weissella, Lactococcus, Bacillus, Enterococcus,* and *Cronobacter* (Zhao et al., 2020). Rice beer varieties such as Apong, Joubishi, and Xaaj of Assam, India, were found to be more abundant in *Lactobacillus, Leuconostoc, Pediococcus, Lactococcus,* and *Weissella* (Das et al., 2019). The similarity in bacterial genera found across the region may be attributed to several factors, such as rice being the most common substrate, fermentation processes, tropical climate, and traditional practices that favor the growth of these microbial communities.

Traditional foods and fermented beverages are increasingly recognized for their health-promoting properties, supported by scientific evidence (Rajkumar et al., 2014; Ivey et al., 2015; Lau et al., 2015; Mota de Carvalho et al., 2018; Şanlier et al., 2019). Consumption of rice beverages is reported to effectively alleviate fatigue, headaches, digestive issues, hypertension, and diabetes (Mishra et al., 2019). Specific microbial strains such as *Lactobacillus* and *Bacillus* are found to be prevalent among ethnic groups of the Tea tribes, Dimasa Kachari, Zeme Naga, and Karbi, which are known for their traditional remedies against jaundice, diarrhea, cholera, urinary disorders, and gastrointestinal ailments (Das A. et al., 2012; Das A. J. et al., 2012; Loying et al., 2024). In addition, the beverages produced by these tribes are rich in nutrients and overexpressing butyric acid, are integral to the daily diet, and are particularly valued for relieving physical fatigue associated with agricultural labor, promoting restful sleep, and aiding digestion (Das A. et al., 2012; Das A. J. et al., 2012; Das et al., 2023).

Several scientific studies have highlighted the predominance of LAB (lactic acid bacteria) such as *Pediococcus, Lactobacillus*, and *Bacillus* in fermented rice beverages, contributing to their nutritional benefits (Deb et al., 2020). *Pediococcus* were highly abundant among the Karbi and Hmar tribes, followed by the Zeme Naga and Tea tribes, exhibiting potential against bile and gastric stresses, hydrophobicity, and colon cell attachment (Jaiswal et al., 2022). Various researchers have also suggested that these strains improve lipid metabolism syndrome induced by hyperlipidemia through modulation of the gut microbiota, impacting metabolic disorders such as type 2 diabetes and obesity (Zhang et al., 2022). Additionally, microbiota isolated from various rice-based fermented beverages demonstrated robust probiotic properties, including bile acid resistance, antibiotic susceptibility, antimicrobial activity, cell surface hydrophobicity, and cell autoaggregation (Giri et al., 2018).

Bacterial diversity plays a crucial role in rice-based fermented beverages. According to our study, the microbial community composition in rice-based fermented beverages may be impacted by the micro-ecological environment present in different production areas, thereby influencing both the diversity and relative abundance of bacteria. The unique mixture of bacteria present in these traditional drinks contributes to their flavor, aroma, and overall quality. Moreover, these microorganisms are responsible for the fermentation process, which not only enhances the beverage’s taste but also extends its shelf life. Therefore, understanding and managing bacterial diversity is essential for maintaining the authenticity and consistency of these cherished ethnic beverages (Zhao et al., 2022).

Alpha diversity analysis suggested higher diversity and abundance of bacteria in the Zeme Naga tribe and Dimasa Kachari tribe than in the Hmar tribe, Karbi tribe, and Tea tribe, which is in agreement with the previous study (Leech et al., 2020). Similarly, beta diversity plots by unweighted UniFrac revealed the similarity of microbial compositions among the Zeme Naga and Dimasa Kachari, followed by the Hmar tribe, Karbi tribe, and Tea tribe, as reported (Atter et al., 2021).

In comparison, genes linked to starch, sucrose, fructose, and mannose were found in the Karbi tribe. The presence of genes in the liver may help in the storage and uptake of glucose, as well as help in carbohydrate oxidation after consuming a meal. Additionally, these genes also act as signal molecules that contribute to metabolic activity, such as hormones producing signals (Laughlin, 2014). However, among the Hmar and Dimasa Kachari tribes, genes associated with oxidative phosphorylation were found, which implies energy production and amino acid utilization and may be related to their different dietary and cultural traditions. The Zeme Naga tribe and Tea tribes displaying an abundance of genes related exclusively to Huntington’s disease raise intriguing questions about the potential genetic factors and susceptibility within these populations. Recent studies have begun to explore the intricate relationship between neurodegenerative diseases, such as Huntington’s disease, and the gut microbiota. Studies have shown that alterations in gut microbiota composition can influence neurological health and may contribute to the pathogenesis of Huntington’s disease (Toledo et al., 2022; Ullah et al., 2023). These findings suggest that the genetic predisposition observed in the Zeme Naga and Tea tribes could be modulated by their unique gut microbiota profiles, thereby affecting the onset and progression of Huntington’s disease. The variations in gene abundance underscore the complex interplay of genetic diversity, microbial ecology, cultural practices, and environmental factors that shape the unique profiles of traditional fermented foods among these diverse tribal communities. Further research is required to unravel the precise mechanisms underlying these intriguing findings and their potential implications for health and microbial adaptation. Recent research on Yucha samples revealed genes linked to chromosome partitioning, nucleotide transport metabolism, lipid transport and metabolism, control of the cell cycle, secondary metabolite biosynthesis and catabolism, and cell motility, which are highly contradictory to our current findings (Zhang et al., 2016). The differences in our findings from Yucha samples may be due to variations in sample origin, dietary practices, or genetic predispositions of the population’s studies in each research effort. Further comparative studies or meta-analyses could help elucidate the factors contributing to these differences and provide a more comprehensive understanding of microbiota functions.

Microorganisms play a vital role in the production of fermented foods by catalyzing the conversion of raw materials into valuable end products. In our study, we have discovered the latent functional capacities of the microbiota as well as diverse microbial populations found in rice-based fermented samples from various ethnic tribes in southern Assam. By discovering these latent microbial functions, our research contributes to a deeper understanding of how microbial communities drive fermentation processes, influencing the nutritional quality and sensory attributes of the final food products. This knowledge not only extends shelf life but also opens avenues for enhancing product quality and fostering innovation in food science (Von Mollendorff et al., 2006). The present study knowledge would help to support the optimization of processing local traditional alcoholic beverage practices, improve product quality and safety, and preserve the cultural heritage associated with these beverages in southern Assam. Future research in this area could further elucidate the complex dynamics at play, potentially leading to advancements in both traditional and novel fermented food production techniques.

To conclude, the present study elucidated the microbial composition of fermented rice-based beverages in southern Assam, including the Zeme Naga, Hmar, Karbi, Dimasa Kachari, and Tea tribes. The study recorded the presence of both harmful and beneficial genera such as *Pediococcus, Lactobacillus, Bacillus, Leuconostoc, Acetobacter, Staphylococcus, Erwinia, Klebsiella, Delftia,* and *Chryseobacterium* and the phyla such as *Firmicutes, Bacteroidetes, Proteobacteria,* and *Actinobacteria*. Notably, the presence of harmful bacteria in the beverages is concerning given the possible health effects among these tribal populations. Therefore, it is imperative to inspect and maintain hygienic practices in these locations intended for ethnic groups.

Further *in vivo* studies are necessary to fully comprehend the significant health implications. This research provides significant insights into the microbial composition of rice-based fermented beverages in southern Assam, shedding light on the traditional fermentation practices and emphasizing their cultural significance and potential health benefits. However, further research is essential to validate these benefits and ensure food safety, contributing to the preservation and safe consumption of these culturally and nutritionally valuable beverages.

## Data Availability

The datasets presented in the study are publicly available. This data can be found here: NCBI BioProject, under accession number PRJNA1017140 (https://www.ncbi.nlm.nih.gov/bioproject/PRJNA1017140/).

## Footnotes

^1^ https://www.bioinformatics.babraham.ac.uk/projects/fastqc/

^2^ https://CRAN.R-project.org/package=vegan
