# Supplementary material for: Metagenomic insights into traditional fermentation of rice-based beverages among ethnic tribes in southern Assam, Northeast India
Source: Front Microbiol. 2024 Sep 11;15:1410098. doi: 10.3389/fmicb.2024.1410098 (PMC11459095; doi:10.3389/fmicb.2024.1410098)
Supplement: Supplementary file 1 [file Data_Sheet_1.docx]

**Supplementary Table 1: Bacterial taxonomic composition data on relative abundance among major taxonomic groups**

**(A- PHYLUM LEVEL)**

|  | Firmicutes | Bacteroidetes | Proteobacteria | Actinobacteria |
| --- | --- | --- | --- | --- |
| HM01 | 88% | 5.30% | 5.90% | 0.50% |
| HM03 | 25.80% | 21.40% | 51.50% | 1% |
| HM05 | 84.20% | 3.60% | 11.70% | 0.50% |
| K101 | 97.00% | 0.00% | 2.90% | 0.00% |
| K102 | 89.60% | 3.10% | 6.90% | 0.30% |
| K104 | 91.30% | 0% | 8.60% | 0.10% |
| ZN01 | 87.20% | 0.80% | 11.90% | 0.10% |
| ZN04 | 51.90% | 5.20% | 32.20% | 10.10% |
| ZN06 | 58.80% | 3.80% | 36.90% | 0.30% |
| DMK01 | 91.90% | 1.10% | 6% | 0.80% |
| DMK02 | 99.60% | 0.10% | 0.40% | 0% |
| DMK03 | 75.60% | 1.50% | 21.20% | 1.70% |
| TT02 | 85.10% | 0% | 14.80% | 0% |
| TT03 | 95.60% | 0.10% | 3.90% | 0.30% |
| TT04 | 99.40% | 0% | 0.50% | 0% |

**(B-CLASS LEVEL)**

|  | Actinobacteria | Bacteroidia | Bacilli | Clostridia | Flavobacteria | Alphaproteobacteria | Betaproteobacteria | Gammaproteobacteria |
| --- | --- | --- | --- | --- | --- | --- | --- | --- |
| HM01 | 0.10% | 5.20% | 80.60% | 7.10% | 0.10% | 0.40% | 0.40% | 5.10% |
| HM03 | 0.90% | 1.90% | 19.90% | 5.70% | 19.10% | 8.10% | 26.80% | 16.50% |
| HM05 | 0.50% | 1.10% | 83.10% | 1% | 1.80% | 9.20% | 0.40% | 2.10% |
| K101 | 0% | 0% | 97% | 0% | 0% | 2.70% | 0% | 0.30% |
| K102 | 0.20% | 2.70% | 86% | 3.40% | 0.20% | 1.20% | 1.30% | 4.40% |
| K104 | 0.10% | 0% | 91.20% | 0.10% | 0% | 8.50% | 0% | 0.10% |
| ZN01 | 0.10% | 0% | 87.10% | 0.10% | 0.80% | 11.40% | 0.10% | 0.40% |
| ZN04 | 10.10% | 4.50% | 42.50% | 8.90% | 0.70% | 11% | 15.20% | 6.10% |
| ZN06 | 0.30% | 0% | 58.40% | 0.40% | 3.20% | 9.70% | 3.90% | 23.30% |
| DMK01 | 0.80% | 0% | 91% | 1% | 0.50% | 4.30% | 0.30% | 1.40% |
| DMK02 | 0% | 0% | 99.60% | 0% | 0% | 0.30% | 0% | 0.10% |
| DMK03 | 1.70% | 0.20% | 75.50% | 0.10% | 1.50% | 1% | 2.80% | 17.40% |
| TT02 | 0% | 0% | 85.10% | 0% | 0% | 14.80% | 0% | 0% |
| TT03 | 0.30% | 0% | 95.30% | 0.30% | 0% | 3.20% | 0.10% | 0.50% |
| TT04 | 0% | 0% | 99.40% | 0% | 0% | 0.30% | 0% | 0.20% |

**S2(C- FAMILY LEVEL)**

|  | Weeksellaceae | Bacillaceae | Staphylococcaceae | Lactobacillaceae | Leuconostoceae | Ruminococcaceae | Acetobacteraceae | Burkholderiaceae | Comamondaceae |
| --- | --- | --- | --- | --- | --- | --- | --- | --- | --- |
| HM01 | 0.10% | 0.10% | 24.10% | 11.80% | 44.50% | 3.40% | 0% | 0% | 0% |
| HM03 | 18.90% | 3.50% | 1.30% | 12.30% | 0.50% | 1.10% | 0.60% | 0% | 20.20% |
| HM05 | 1.70% | 1.70% | 0.10% | 80.10% | 0.70% | 0.40% | 7% | 0% | 0.10% |
| K101 | 0% | 0% | 0% | 93.50% | 3.40% | 0% | 2.60% | 0% | 0% |
| K102 | 0.20% | 0.60% | 0% | 84.20% | 0.90% | 1.70% | 0.40% | 0% | 0.30% |
| K104 | 0% | 0.20% | 0% | 91% | 0% | 0% | 8.30% | 0% | 0% |
| ZN01 | 0.80% | 12.40% | 0% | 62.70% | 11.90% | 0% | 11.30% | 0% | 0% |
| ZN04 | 0.70% | 1.40% | 1.30% | 32.90% | 0% | 6.10% | 0.50% | 14.30% | 0.30% |
| ZN06 | 3.20% | 6.10% | 0.10% | 51.40% | 0.40% | 0.10% | 0.60% | 0% | 1.50% |
| DMK01 | 0.50% | 9.30% | 2% | 69.40% | 0.40% | 0.10% | 2.80% | 0% | 0.10% |
| DMK02 | 0% | 0.10% | 0% | 99.50% | 0% | 0% | 0.10% | 0% | 0% |
| DMK03 | 1.50% | 42.60% | 1.20% | 26.30% | 3.90% | 0% | 0.60% | 0% | 1.20% |
| TT02 | 0% | 0% | 0% | 84.70% | 0.30% | 0% | 13.80% | 0% | 0% |
| TT03 | 0% | 66.50% | 1.20% | 23.90% | 3.40% | 0% | 3.10% | 0% | 0.10% |
| TT04 | 0% | 0% | 0% | 97.60% | 1.80% | 0% | 0.20% | 0% | 0% |

**S2 (D- GENUS LEVEL)**

|  | Chryseobacterium | Bacillus | Staphylococcus | Lactobacillus | Pediococcus | Leuconostoc | Acetobacter | Delftia | Erwinia |
| --- | --- | --- | --- | --- | --- | --- | --- | --- | --- |
| HM01 | 0.10% | 0.10% | 24.10% | 0.80% | 11.00% | 0.90% | 0% | 0% | 0.90% |
| HM03 | 18.90% | 3.30% | 1.20% | 5.90% | 6.30% | 0% | 0.50% | 19.60% | 5.10% |
| HM05 | 1.70% | 1.70% | 0.10% | 38.40% | 40.70% | 0.10% | 6.30% | 0.10% | 0.20% |
| K101 | 0% | 0% | 0% | 26.90% | 64.60% | 3.30% | 2.40% | 0% | 0% |
| K102 | 0.20% | 0.60% | 0% | 6.30% | 77.90% | 0.10% | 0.30% | 0.20% | 1% |
| K104 | 0% | 0.20% | 0% | 18.70% | 71.20% | 0% | 8.20% | 0% | 0% |
| ZN01 | 0.50% | 11% | 0% | 23.90% | 37.80% | 10.80% | 10.30% | 0% | 0% |
| ZN04 | 0.70% | 0.80% | 1.30% | 29% | 3.90% | 0% | 0.50% | 0.30% | 0% |
| ZN06 | 3.20% | 5.90% | 0.10% | 6.20% | 44.60% | 0% | 0.50% | 1.10% | 4.40% |
| DMK01 | 0.50% | 6.20% | 1.90% | 35% | 33.90% | 0% | 2.50% | 0.10% | 0.10% |
| DMK02 | 0% | 0.10% | 0% | 97.70% | 1.70% | 0% | 0.10% | 0% | 0% |
| DMK03 | 1.50% | 42.40% | 1.20% | 5.60% | 20.60% | 1.30% | 0.50% | 1.10% | 6.20% |
| TT02 | 0% | 0% | 0% | 51.20% | 32.10% | 0.10% | 12% | 0% | 0% |
| TT03 | 0% | 64.40% | 1.20% | 23.10% | 0.80% | 0.10% | 1.90% | 0% | 0.20% |
| TT04 | 0% | 0% | 0% | 95.20% | 2.30% | 0.30% | 0.20% | 0% | 0% |

| Tribes | Local beverage name | Ingredients used | type of rice |
| --- | --- | --- | --- |
| Zeme Naga | **Zao** | Rice and sprouted rice (*Oryza sativa L.*) | Local sticky rice/glutinous rice |
| Dimasa Kachari | **Judima** | Rice and bark plant of thempra (*Acacia pennata*) | Local sticky rice/glutinous rice |
| Hmar | **Zu** | Rice and bark of Zangzu plant (*Acacia pennata*) | Local sticky rice/glutinous rice |
| Karbi | **Hor Alank** | Rice and leaves of marthu *arvo* (*Croton joufra)* | Local sticky rice/glutinous rice |
| Tea tribe | **Haria** | Rice and bark of Yastimadhu (*Glycyrrhiza glabra)* | Local sticky rice/glutinous rice |

**Supplementary figure 1: Details of the rice-based fermented beverages for each distinct ethnic groups**
